# Imaging of nanoparticle dynamics in live and apoptotic cells using temporally-modulated polarization

**DOI:** 10.1038/s41598-018-38375-9

**Published:** 2019-02-07

**Authors:** Omer Wagner, Moty Schultz, Eitan Edri, Rinat Meir, Eran Barnoy, Amihai Meiri, Hagay Shpaisman, Eli Sloutskin, Zeev Zalevsky

**Affiliations:** 1 Faculty of Engineering and the Institute for Nanotechnology and Advanced Materials, Bar-Ilan University, Ramat-Gan, 5290002 Israel; 2 Department of Physics and the Institute for Nanotechnology and Advanced Materials, Bar-Ilan University, Ramat-Gan, 5290002 Israel; 3 Department of Chemistry, Institute for Nanotechnology and Advanced Materials, Bar-Ilan University, Ramat-Gan, 5290002 Israel

## Abstract

Gold nanoparticles are widely exploited in phototherapy. Owing to their biocompatibility and their strong visible-light surface plasmonic resonance, these particles also serve as contrast agents for cell image enhancement and super-resolved imaging. Yet, their optical signal is still insufficiently strong for many important real-life applications. Also, the differentiation between adjacent nanoparticles is usually limited by the optical resolution and the orientations of non-spherical particles are unknown. These limitations hamper the progress in cell research by direct optical microscopy and narrow the range of phototherapy applications. Here we demonstrate exploiting the optical anisotropy of non-spherical nanoparticles to achieve super-resolution in live cell imaging and to resolve the intracellular nanoparticle orientations. In particular, by modulating the light polarization and taking advantage of the polarization-dependence of gold nanorod optical properties, we realize the ‘lock-in amplification’, widely-used in electronic engineering, to achieve image enhancement in live cells and in cells that undergo apoptotic changes.

## Introduction

Biomarkers and contrast agents are frequently used in current day imaging to mark specific locations, increase the imaging signal and maximize the signal to noise ratio (SNR)^1^. Gold nanoparticles (GNPs) are good candidates for that purpose, as they are highly biocompatible, do not suffer from photobleaching and are particularly-well fitted to optical imaging due to their high scattering/absorbance cross section in the visible spectrum^2–4^. GNPs are also associated with surface plasmon resonance (SPR), which is generated from collective oscillations of free conduction electrons, induced by the electromagnetic field illumination^5^. The SPR is responsible for the GNP high absorption and scattering cross sections at specific spectral peaks and also enhances its response to near-field electromagnetic fields^6^. These attributes are applied to probe many biomolecular interactions such as Biotin-streptavidin, Antibody-Antigen, Toxin-Receptor^6–9^. Importantly, the surface plasmon resonance (SPR) is tunable by design of the GNP shape, size and coating^10,11^.

The geometrical shape of a GNP also plays an essential rule in the biomarker functionality, because it directly influences the interaction with the sample. While spherical GNP tracers are valuable for sample characterization, an even more detailed and unique information may be revealed by employing non-spherical GNPs. For example, the crossing of cellular membranes by the nanoparticles, occurring by either a direct membrane penetration or by a membrane-wrapping mechanism, strongly depends on particle shape and orientation^12,13^. Such interactions determine the extent of the cellular nanoparticles uptake in targeted drug delivery - shape and size have the potential to increase uptake into a desired cell type while minimizing the uptake into the other cell types^14^. Indeed, special modes of wrapping, reorientation, and transport were predicted for ellipsoidal, cylindrical and rod-like nanoparticles^12,15,16^. Even more complex particle transport dynamics are possibly taking place during gradual cell apoptosis^17–20^.

Currently, the existing experimental methods do not allow the unstained cellular compartments to be imaged in real life, with a high resolution, in parallel with the detection of nanoparticle positions and orientations. The development of such methods should provide a deeper and more detailed understanding of the apoptosis, as also of many other vital biological processes.

The main limitation on conventional optical imaging systems is that they may not produce an adequate SNR in real case environments, which are noisy and require diluted concentrations. Moreover, the nanoparticle sizes are typically smaller than the imaging wavelength, in which case the classical optical resolution does not allow the spatial orientation of the GNP to be resolved. Many approaches to increase the SNR have been reported. In particular, background reduction by hyperspectral imaging^21,22^, marker fluorescence or Raman response enhancement by SPR^23,24^, intensity-modulated GNP flickering^25^, and several other methods were employed. Yet, most of these approaches require dedicated sophisticated and expensive equipment. Also, these methods do not exploit the effect of the spatial orientation of the nanoparticles on the optical response as discussed below. This effect potentially allows for a further increase of the SNR and can reveal the GNP orientations.

To induce the SPR, the illuminating electromagnetic field must have its irradiance and polarity at a specific direction with respect to the GNP^5^. For example, a light beam incident onto a 300 nm-high flat triangle, would give rise to two different scattering eigenmodes, depending on the angle of incidence being either 15° or 75°. Keeping the angle of incidence constant, but changing between the transverse and the longitudinal polarity would produce different modes as well^26^. Many types of nanoparticle geometries have reached industrial production level through chemical synthesis or lithography processes. Remarkably, the SPR response is, in general, different for each of these geometries. Among the examples that were extensively researched are the nanocubes^27^, the nanostars^28–30^, the popcorn-like nanoparticles^31,32^, the nanotriangles^26,33,34^, the bipyramids and the gold nanocrescents^6^. Yet, arguably, the simplest GNPs with an anisotropic SPR are the gold nanorods.

Here we take advantage of the fact that the scattered light from an anisotropic GNP, such as a gold nanorod (GNR), depends on both the transverse and the longitudinal SPR excitation modes. We selectively excite these modes by varying the polarization of the illuminating beam^35–37^. Alternatively, we selectively image these modes, by separating the detected light by polarization, with an analyzer introduced in front of the light detector.

Moreover, we exploit this polarization-selective excitation and detection to modulate the signal, employing an otherwise-conventional bright-field (BF) or dark-field (DF) imaging setup. With the GNRs used as the labels, polarization modulation gives rise to a temporal flickering of the signal. In this manner, the GNRs can serve as high contrast markers for polarization imaging methods based on lock-in amplification, as in the differential polarization microscopy^38–40^. Because the anisotropy ratios of most biological specimens are small, ranging between 10^−2^ to 10^−5^ ^40^, the GNR-enhanced signal is much stronger than in the previous particle-free implementations^41^. Thus, high quality imaging is possible. Furthermore, we take advantage of the polarization modulation not only to increase the SNR but also to obtain the lock-in phase. This phase is connected to the GNR orientation, so that a map of rod positions and orientations is obtained, providing a valuable tool for the research of intracellular nanoparticle dynamics. Adjacent rods that differ in their positions are also separately resolved, enhancing the resolution^42^. In particular, we demonstrate a simple approach to use the selective excitation of the SPR modes to increase the robustness of the imaging, otherwise limited to only highly-intense illumination settings, focused confocal laser scanning or two-two-photon microscopy.

Other eccentric shapes such as nanostars or popcorn nanoparticles could be used to enhance the method even further. For example, the GNR orientation derived using a rod is its projected angle on the 2D imaging plane. As reported in ref.^30^, nanostar particles exhibit multiple different SPR wavelengths, corresponding to different 3D-orientations of light polarization. Using our method to obtain the 2D-projection of the particle for each of the SPR wavelengths, would allow the full 3D orientation of the particle to be resolved. Additional enhancement of the SNR can be achieved by staining the GNR with fluorophores to invoke plasmon-enhanced fluorescence or plasmon-enhanced Raman response^23,24,43^.

For simplicity and as a proof of concept, we apply this polarization-modulated lock-in imaging (PMLI) method with simple GNR particles. First, we apply the method on few test samples, where positions and orientations of individual GNRs were ascertained by SEM. Then we apply the same techniques to the live cell imaging, exposing the intracellular nanoparticle dynamics difference between normal cells and cells that undergo apoptotic changes.

## Results

**Figure 1:**
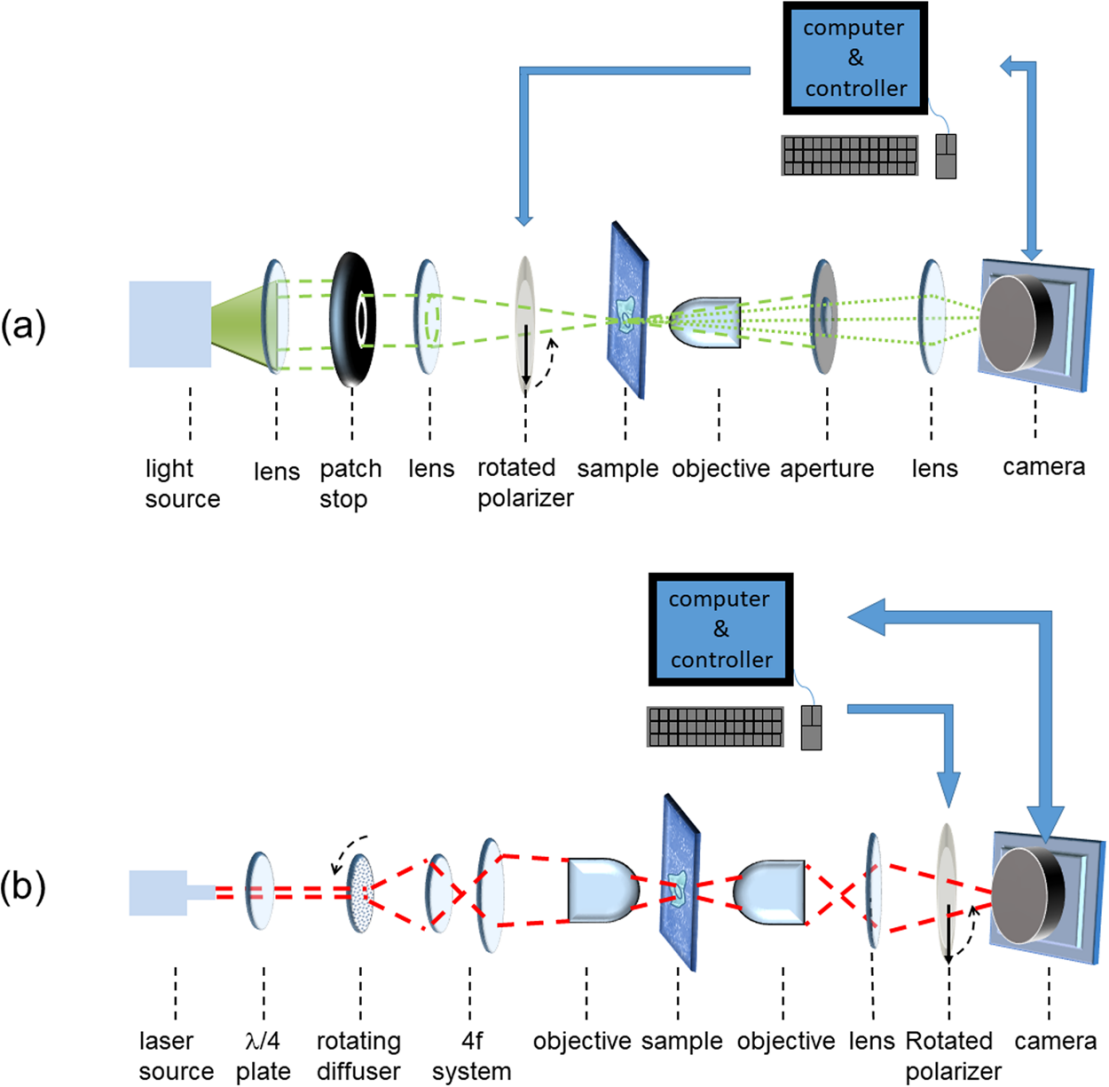
Optical setup. A dark field imaging system (**a**), incorporates a rotated polarizer, which is positioned between the condensing lens and the sample. Polarizer rotation induces signal flickering, allowing signal amplification by the lock-in technique. A bright field imaging system (**b**), incorporates a λ/4 plate, creating a circularly-polarized light. Here, the rotating polarizer is located between the tube lens and the camera.

To test the validity of the PMLI method, we first employ it for the optical visualization of a few individual GNRs (diameter ≈ 50 nm; length ≈ 145 nm) dispersed on a cover slip (see Methods). We chose the illumination wavelengths as 780 nm and 530 nm, matching the transverse and the longitudinal SPR excitation modes, respectively. By modulating the illumination polarization, a temporal flickering of the GNR signal was induced. This flickering was then used to amplify the signal by the lock-in technique (see Methods). In principle, either a DF or a BF setup may be employed (Fig. 1).

The DF imaging is, arguably, the most suitable imaging method for particles of this size, where no additional labeling is employed^44^. Also, the DF has been previously employed, among a few other imaging modalities^45,46^, to detect the orientations of individual particles deposited on a bare slide^35^. However, in spite of these advantages of the DF imaging, we employ here the BF imaging, as well. Although the BF is typically less efficient than the DF in terms of the SNR, the BF imaging depends on light absorbance, rather than scattering, thus potentially providing an additional sample characterization. Furthermore, the BF also employs a less complicated hardware and may be easier to combine with the existing lab equipment.

**Figure 2:**
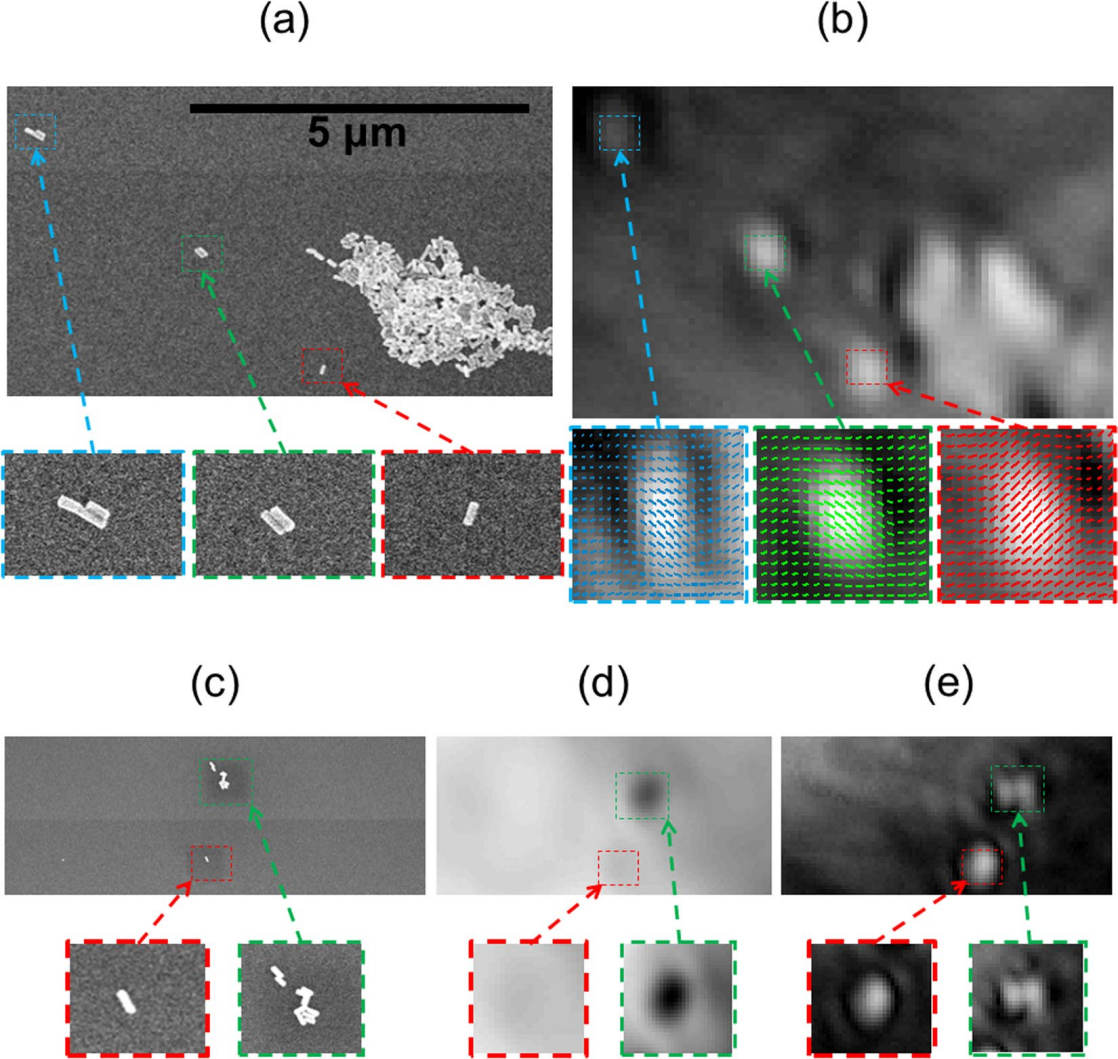
The polarization-modulation method resolves nanoparticles and their orientations. Imaging the same area of the sample by both SEM (**a**) and our BF polarization-modulation method (**b**), demonstrates the detection of individual nanoparticles by our method. Magnified images of GNRs, shown below, correspond to the locations marked by colored rectangles in the main panel. The calculated GNR orientations are illustrated by short lines, overlaid on top of the magnified GNR images in section (**b**). Yet another sample spot is sampled by SEM (**c**), conventional non-modulated BF (**d**), and polarization-modulated (PMLI) BF (**e**). To image the same spot by all the three techniques, its location was determined by a special TEM grid. The optical image intensities are normalized, to allow for their mutual comparison. Note the far better resolution and contrast of the PMLI method.

As a stringent control of the PMLI, we compare it with the SEM images of exactly the same area (Fig. 2(a)). Note the remarkable enhancement of the optical signal by polarization modulation, allowing the individual GNRs to be clearly distinguished in *I(x*,*y)* images obtained in a BF mode (Fig. 2(b)). We zoom-in onto a few spots across the imaged area, where GNRs are located (bottom images in Fig. 2); note the identical spots in SEM (Fig. 2(a)) and optical microscopy (Fig. 2(b)), marked with the same colors.

The advantage of PMLI is not limited to just the SNR enhancement. Rather, the PMLI also provides the relative rod orientations, which are obtained from the phase of the flickering signal. We show the calculated GNR orientations by rod symbols in the magnified images (Fig. 2(b), bottom). To obtain the absolute orientations, we calibrated the flickering phase based on the SEM-determined orientation of a single rod. Next, the absolute orientations of all the other rods were resolved, demonstrating an excellent agreement with the SEM (Fig. 2(b)). The agreement achieved between the SEM and the PMLI, emphasizes the capability of our technique to correctly resolve the GNR orientations.

In addition to resolving the GNR orientations, our polarization-modulation lock-in amplification technique also improves the separation between the imaged features and the visibility of nano-objects. We demonstrate this fact in Fig. 2(c–e), where the same area of the sample is shown, imaged by SEM (c), by an ordinary BF microscopy with no polarization modulation (d), and by the PMLI BF method (e). The magnified views on two GNR locations appear in the insets, marked by matching colors for all the three imaging methods. In particular, an individual, well-separated, GNR appears in the area marked by a red rectangle. While it is hardly more than a faint blur in the conventional BF image (d), it is clearly resolved by the polarization modulation (e), demonstrating significant resolution enhancement. Note the color inversion in this image. The GNR absorbs the light and therefore appears darker than its background by the conventional BF imaging. Yet, the GNR appears bright in PMLI, as the intensity is ramped up for only those features which flicker at the right frequency.

A further demonstration of resolution enhancement is provided by the area marked in green (in Fig. 2(e)), which contains two elongated clusters, spaced by approximately 150 nm. The PMLI clearly resolves between them, far surpassing the diffraction limit of the 780 nm wavelength illumination, while only a single dark shadow is seen by the conventional BF imaging. With that, the complex shape of these GNR clusters somewhat complicates the modulation imaging. In particular, the cluster orientation is not detected correctly. The same issue occurs for the large cluster in Fig. 2(a,b). Apparently, the SPR properties of the GNRs are changed by the close mutual proximity of GNRs within the clusters. A further research is clearly called for, to fully understand and overcome this issue.

**Figure 3:**
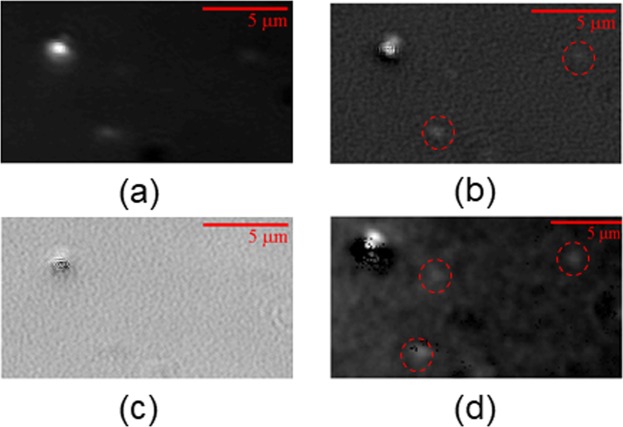
Dark-field phase-modulated imaging of GNR particles enhances sensitivity and resolution. (**a**) A low resolution image, obtained by the conventional dark-field microscopy. (**b**) Applying lock-in detection, with the phase matched to that of the GNR, allows the individual GNR at the bottom of the image to be resolved. (**c**) Only the feature in the left upper corner of the image is visible, when the lock-in detection is applied with an opposite phase. Fitting the theoretical polarization-dependent reflectance to the experimental pixel intensities (shown in panel (**d**)), enhances the revealed GNRs’ signals (marked by dashed red circles).

The proposed method of polarization modulation is quite general, applying beyond the BF imaging mode. In particular, we demonstrate here the implementation of this method for the DF imaging, combining the advantages of both techniques. We image the same field by the conventional DF method and by the PMLI DF microscopy. The conventional DF method is unable to resolve several individual GNRs, scattering weakly below the noise level of the system: the central part and the right side of the field, where these particles are located, are almost totally dark in Fig. 3(a). When the lock-in processing is applied to the polarization-modulated imaging, the differentiation between the oscillating signal of the GNRs and the random noise of our experimental setup is successful (Fig. 3(b)). Applying the lock-in method to inspect only the out-of-phase data (Fig. 3(c)) eliminates the single GNRs’ signals, hinting that all the three separate rods have the same orientation. Yet, parts of the intense scattering spot, at the upper left corner of the image, are still visible. This fact may be resulted by multiple closely-located GNRs, with their SPR modified by mutual proximity, as discussed above. A further insight may be obtained by fitting a sine function to the temporal dependence of the intensity of each pixel I_sig_(x,y,t). The fitted individual pixel amplitudes are shown in Fig. 3(d); note the three individual GNRs (marked by red circles), which are clearly resolved.

Many properties of the GNR, such as its friction coefficient or its absorption spectrum, are rotationally-anisotropic. When a GNR is used as a tracer particle to probe the surrounding physical or biological medium, knowing its orientation is essential, for the interpretation of its dynamics in terms of the medium properties to be correct. For GNRs employed in phototherapy, the method’s efficiency depends on the relative orientation of the GNRs with respect to the polarization of the illuminating radiation. With the particle orientation known, the polarization may be optimized, minimizing the risks and maximizing the efficiency of these therapies. To further demonstrate the exciting capabilities of the PMLI, we apply it to live biological cells, enhancing the visibility of intracellular structures and following the unexpected orientation dynamics of the intracellular GNRs.

**Figure 4:**
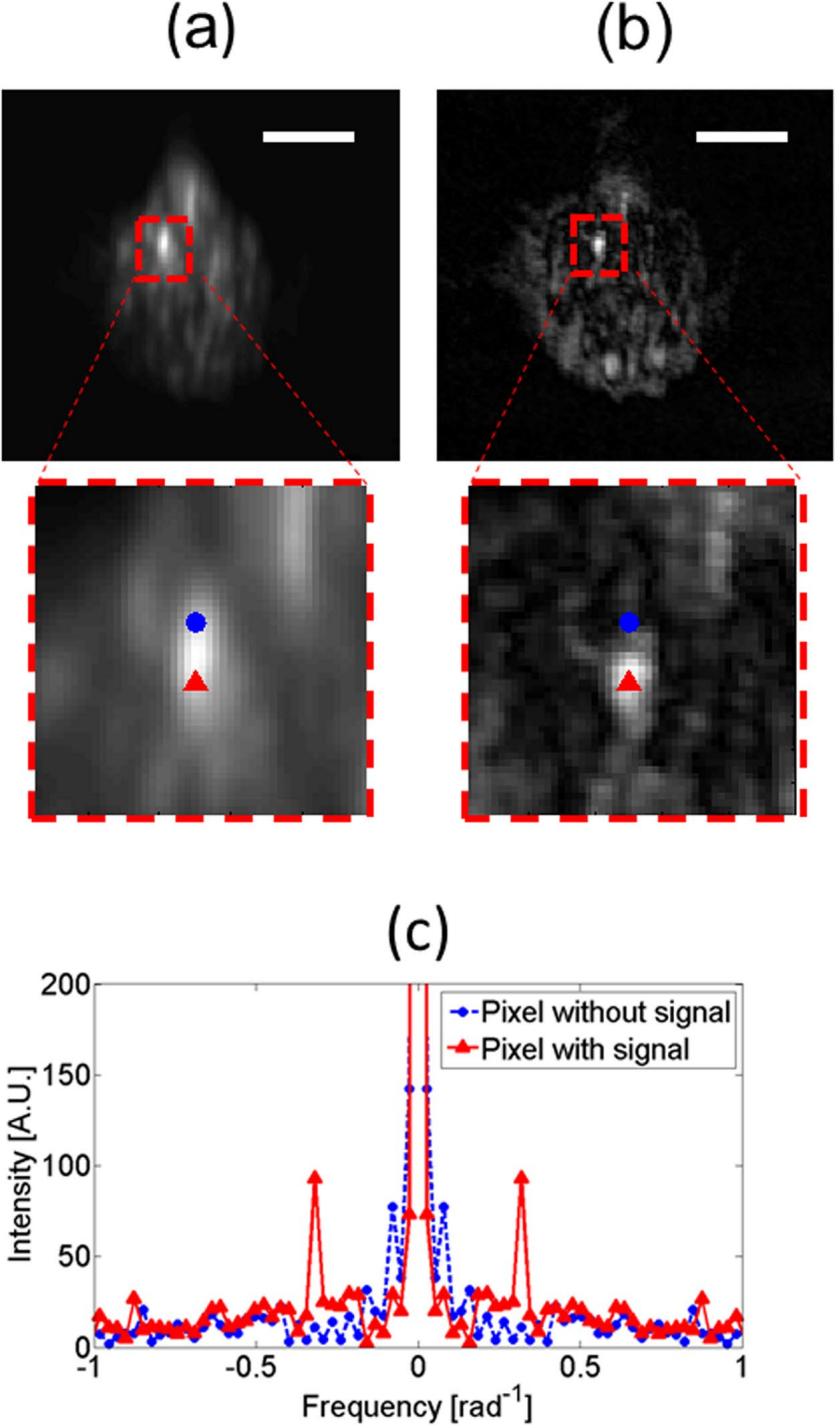
Fixed B-16 cells, labeled by GNRs, demonstrate an enhanced resolution when imaged by PMLI in the DF mode. (**a**) A conventional DF imaging yields a blurry image of a fixed B16 cell, in spite of it being labeled by the GNRs. (**b**) The image is significantly sharpened and its SNR is improved by the DF PMLI method. The scale bar lengths (white) correspond to 10 μm. The bottom images zoom-in onto the field of view marked by a red dashed rectangle. Remarkably, we demonstrate here the capability of the PMLI to separate between two adjacent pixels (**a** red triangle and a blue dot), in spite of them being much closer together than the optical resolution limit. The red pixel is blinking at the modulation frequency (solid red curve in (**c**)), hinting it to be a true scattering from a GNR. The blue pixel does not blink at the same frequency (dashed blue curve in (**c**)), indicating it to be induced by the environmental noise.

First, we carry out the measurements on fixed B-16 cells, labeled by GNRs, implementing the PMLI method in the DF imaging mode. A conventional DF image is shown in Fig. 4(a), where the cell is blurry. The sharpness dramatically improves when the PMLI is applied (see Fig. 4(b)). In the magnified images (in dashed red rectangles, below the main panels), we mark two neighboring pixels: one of these demonstrates a GNR signal (red dot), while the second one does not include any significant GNR contribution (blue dot). The frequency response of both the data pixel and the no-data pixel is shown in (c). A sharp peak at the 1/π frequency is clearly observed for the pixel with the GNR signal, as expected for a response of a GNR. The pixel without the GNR signal contribution does not contain such a peak, further supporting the validity of our analysis.

Applying PMLI on a different batch of fixed B16 cells in BF mode shows similar results, shown on the Supplementary Information SI-1.

**Figure 5:**
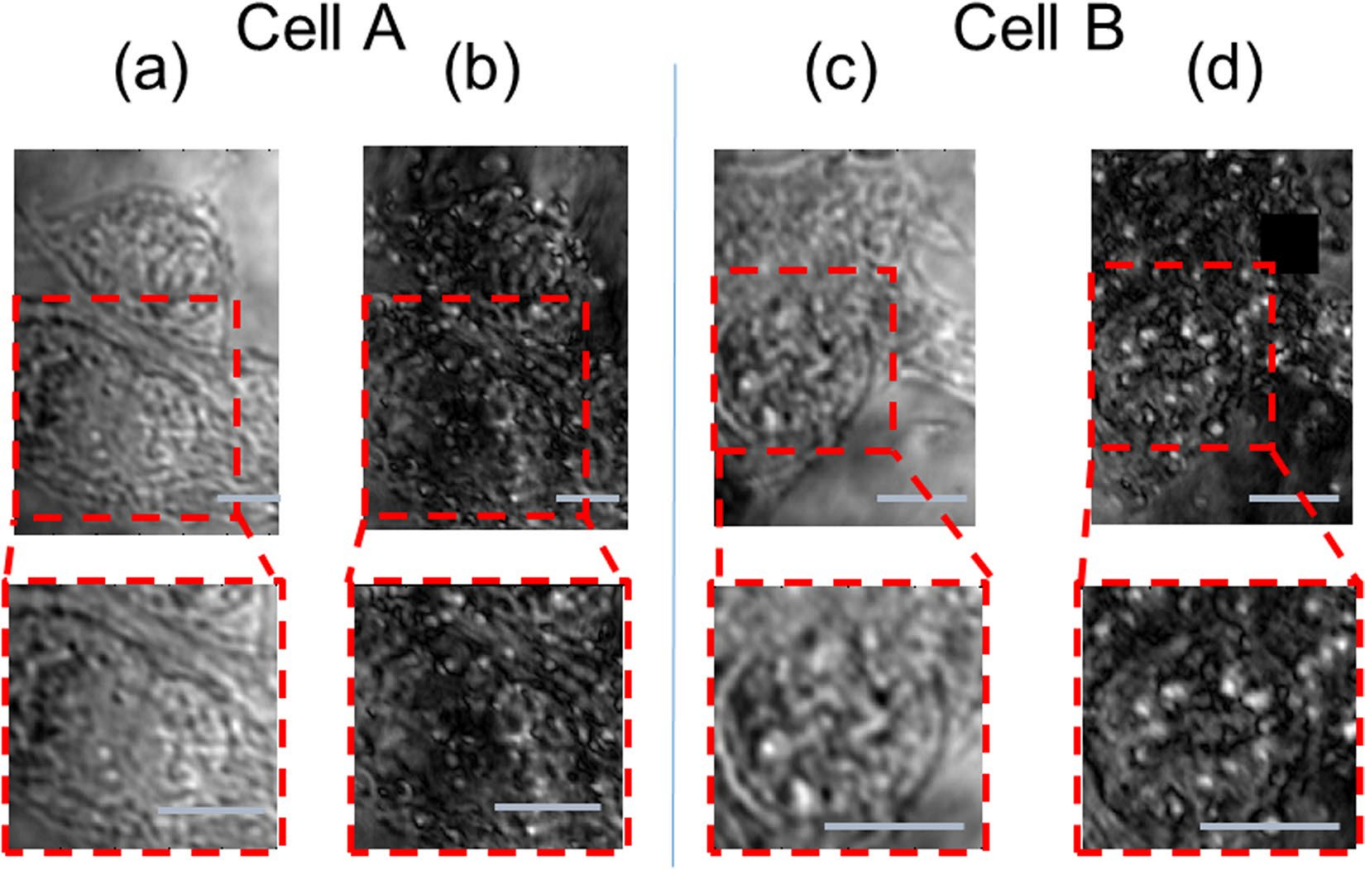
The contrast and the resolution of bright-field images of GNR-free live cells are enhanced by PMLI. The images demonstrate that a much sharper contrast is achieved with the PMLI method. Two cells are shown, the cells are imaged without (**a**,**c**) and with (**b**,**d**) the application of the PMLI, for the sake of comparison. The same field of view is chosen in (**a**) and in (**b**); another field of view is shown in (**c**,**d**). The areas magnified in the bottom panels, are marked by red rectangles in the lower-magnification images on top. The intensity values are normalized, to allow for a fair comparison between the images. The scale bar lengths (gray) correspond to 10 μm.

Next, we apply our method to living MC38 cells, as a control for our studies of apoptosis-undergoing cells, which are presented in the following paragraphs. Thus, for now, the apoptosis process is not initiated, and the cells reside in DMEM (see Methods). Typical images of GNR-loaded cells are shown in Fig. 5 (images of a GNR-free control group are shown in the SI). Two cells are shown here, each is imaged by both the conventional BF imaging (a, c) and by the PMLI (b, d). A significantly better contrast and a higher SNR is achieved with the PMLI technique.

**Figure 6:**
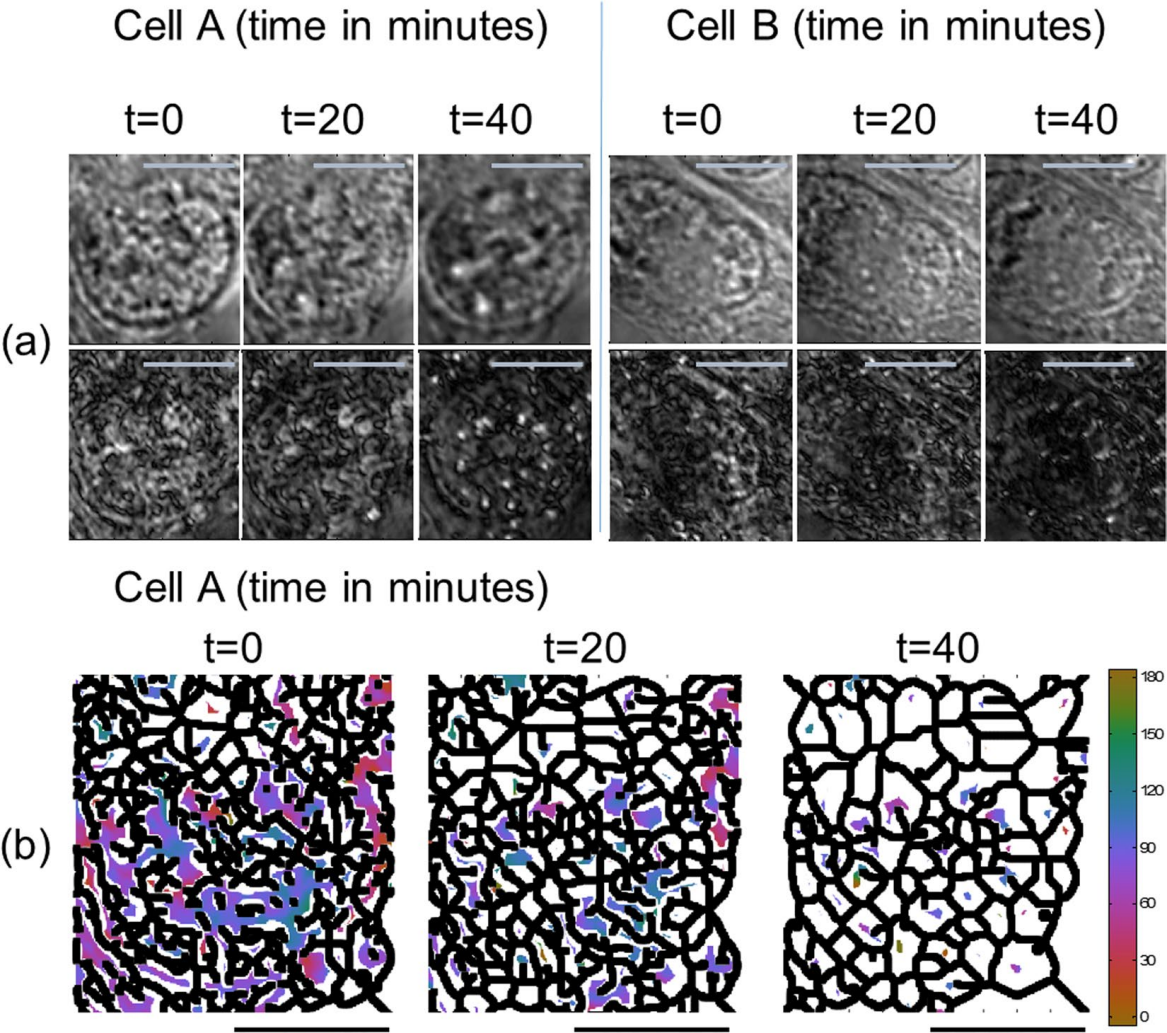
PMLI, carried out in the BF mode, demonstrates a temporal variation of GNR orientations to take place in live GNR-loaded cells. (**a**) The bright-field images (top row), do not disclose any significant temporal post-loading variation in cells’ structures. However, such post-loading changes are clearly visible by the polarization modulation imaging (bottom row), which is sensitive to a local optical anisotropy within the samples. In particular, the fine pattern of intracellular black curves (bottom row), coarsening as a function of time, suggests that the distribution of GNR orientations is time-dependent and non-random. The observed time-evolution of GNR-loaded cells may have an impact on their biological behavior, yet remains unnoticed by the conventional imaging methods. The imaging times, post-loading, are labeled on top. (**b**) The temporal evolution of the intracellular GNR orientations, following the GNR loading into the cell, exhibits a coarsening of the GNR orientation domains, unnoticed by the conventional imaging methods. The black boundaries separate between distinct strong intensity areas in the PMLI images. A threshold on the intensity was employed to remove the background noise. Pixels below the threshold appear in white. The color map (in angle degrees) corresponds to the GNR orientations. Scale bars in both (**a**,**b**) correspond to 10 μm. The same raw data are used here, as for the cell A in (**a**).

The PMLI, even when assisted by GNR-loading into the cells, is by no means limited to static situations only. To demonstrate the capabilities of this method for the dynamic processes, we follow intriguing temporal changes occurring in the GNR-loaded cells, at longer post-loading times (see Fig. 6). Importantly, none of these changes are discernible by the conventional BF or DF imaging, as these methods do not provide any information as for the GNRs intracellular orientations. A typical time variation of GNR orientations is demonstrated in Fig. 6(a), for the same two cells which appear in Fig. 5 (Cell A and B). The top row shows the conventional BF images, while the PMLI results appear at the bottom. The three columns correspond to different post-GNR-loading times, 20 minutes apart (see labels). Note the fine pattern of black curved intracellular boundaries, visible in the modulated polarization images at short post-loading times. This pattern seems to correspond to tiny intracellular regions or compartments, each having a different GNR orientation. Remarkably, this pattern coarsens at longer times, suggesting the growth of GNR orientation domains, unnoticed by the conventional imaging techniques.

To further emphasize the dynamics of the GNR orientation domains, we apply a threshold on the modulated polarization imaging intensity, removing all intensity values below 30% of the maximal value. Then, the morphological skeletonization is applied to the remaining intensity data, retrieving the boundaries which separate between the zero-intensity regions. The resulting domain boundaries exhibit a clear coarsening as a function of time, as demonstrated in Fig. 6(b), where the boundaries are shown in black and the colors inside the domains represent the local GNR orientation. The colors are only shown for the domains where the total intensity was above the 30% threshold. The precise biological processes behind the observed time evolution are yet to be identified. However, we anticipate this time-evolution of the intracellular GNR distributions, unnoticed by the conventional BF imaging, to possibly have an impact on the behavior of nano-rod-treated cells, with potential far-reaching consequences in cell research and therapy.

**Figure 7:**
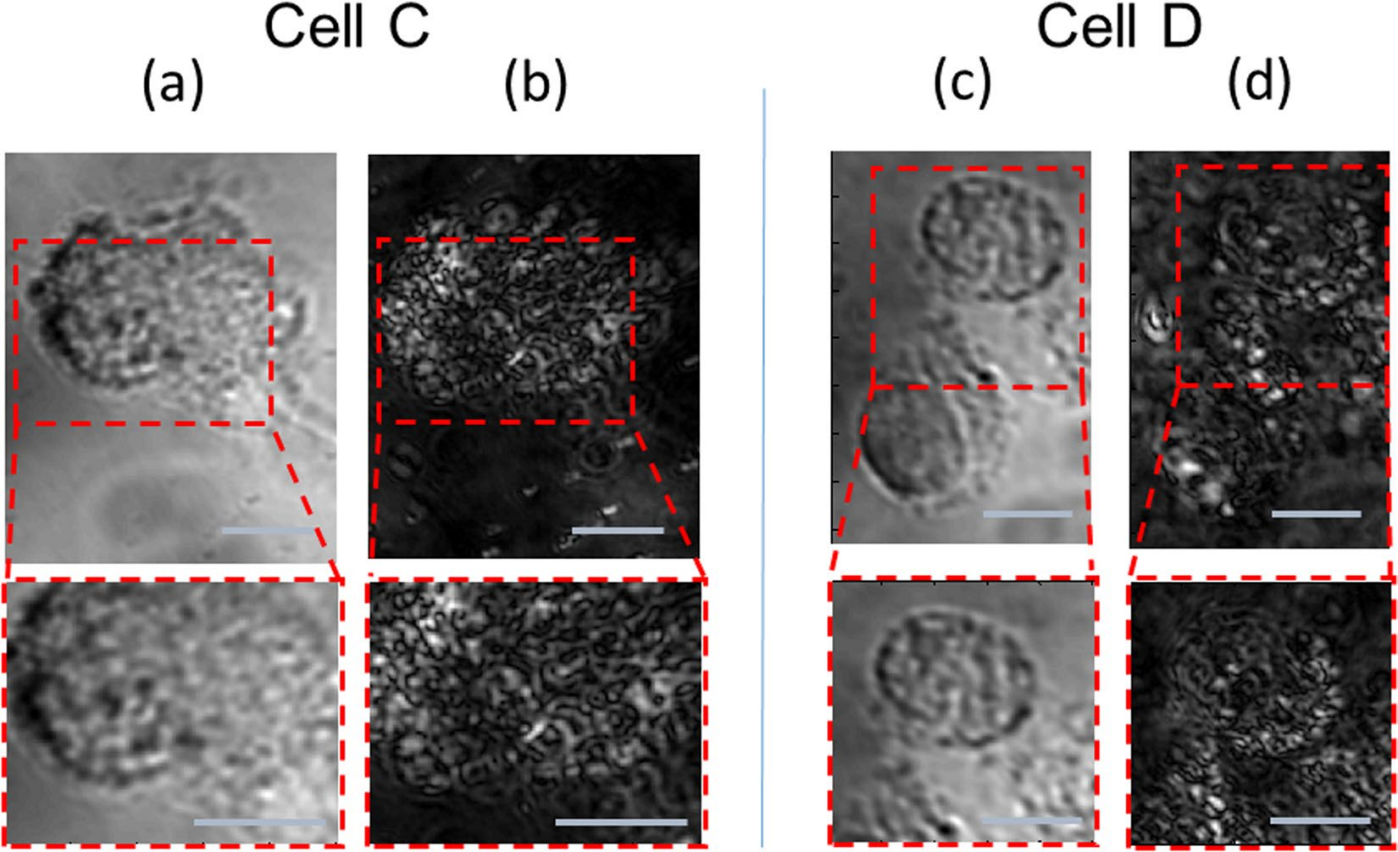
Live GNR-treated cells, undergoing an apoptosis process, resolved by the PMLI BF imaging. Two typical cells images of the same area, without (**a**,**c**) and with (**b**,**d**) the PMLI application. Note the fine intracellular details, visible by the PMLI microscopy. The insets at the bottom zoom onto the area marked by the dashed red rectangle on top. The intensity values are normalized, for a fair comparison between the different images. The scale bars correspond to 10 μm.

To ascertain that the observed post-loading dynamics in live cells is not a result of a possible early stage of an apoptosis, we intentionally induce the cells’ apoptosis and follow the apoptotic transformations by the PMLI BF imaging. For the induction of apoptosis, H_2_O_2_ is introduced into the medium (see Methods); the subsequent temporal evolution of the cells was followed by microscopy. In Fig. 7 we show the imaging of two typical cells started 3 and 5 hours after the initiation of the apoptosis respectively. At that time, the cells clearly show the classic signs of cell death, and specifically the shrinkage of the cell body and nucleus typical for the early apoptosis^17–20^. Panels (a) and (c) show the conventional BF imaging, without the polarization modulation, while (b) and (d) show the PMLI images. Remarkably, the contrast of the PMLI images is comparable to that of the non-apoptotic GNR-loaded cells (Fig. 5); no significant contrast loss is resulted by the apoptosis.

**Figure 8:**
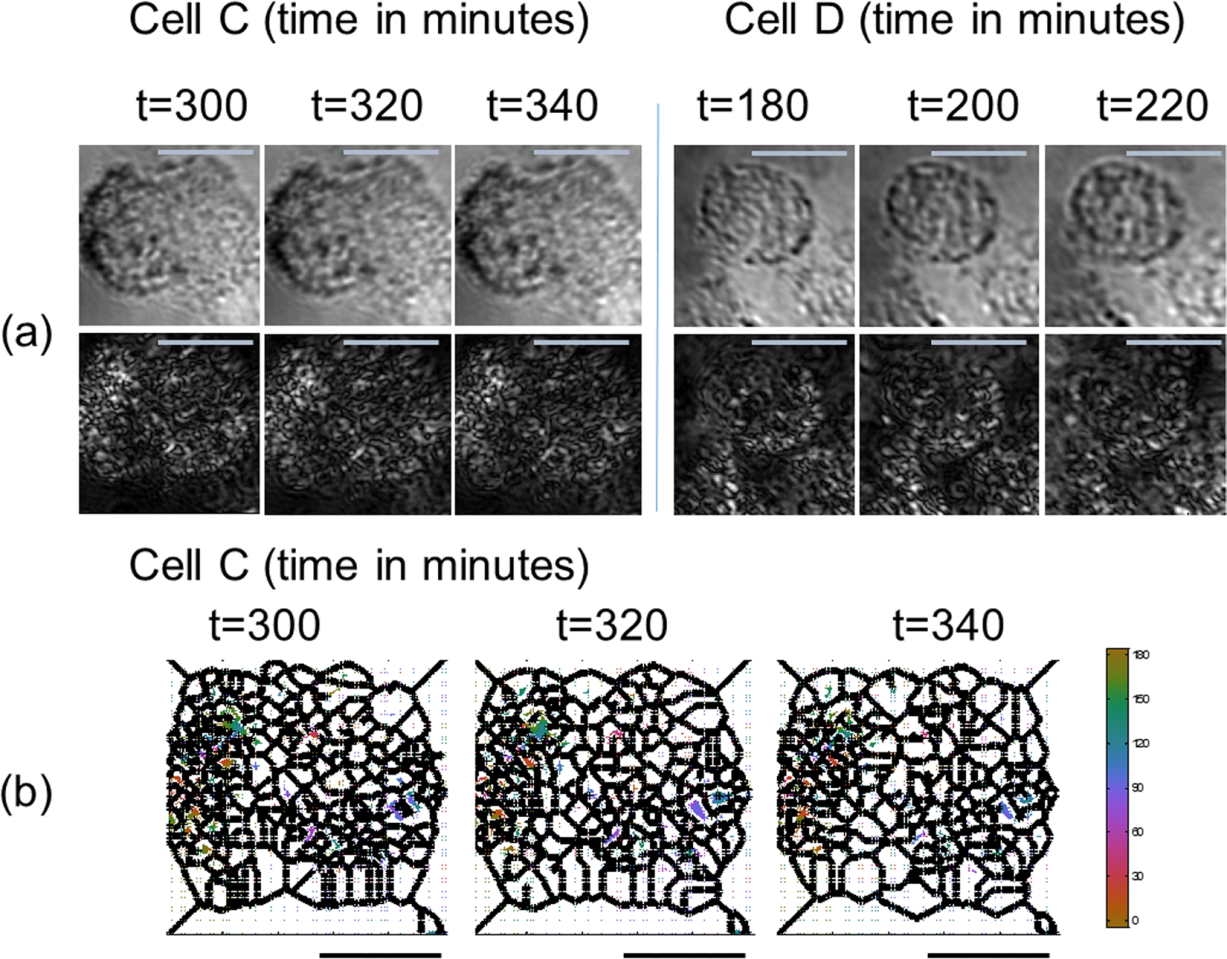
Bright-field images of dynamics in live GNR-treated cells, undergoing an apoptosis process, resolved by the PMLI BF imaging. In row (**a**), two typical cells images of the same area with the three columns for each cell representing images taken at different times (see labels on top). The conventional BF imaging is demonstrated in the top row; the PMLI images for the same time points are shown at the bottom. The dynamics of GNR orientations during the apoptosis of cell C are visualized in row (**b**). A threshold on the GNR intensity was employed to eliminate the background noise, pixels below the threshold appear in white. The color map corresponds to the GNR orientation angles in degrees. The curved black boundaries separate between domains with strong modulated polarization signal intensities. The scale bars, in both (**a**,**b**) correspond to 10 μm.

At later times during the apoptosis, the PMLI reveals significant changes in intracellular GNR orientations (Fig. 8 rows (a) and (b)). However, the behavior is very different from that of the live GNR-loaded cells in Fig. 6. In particular, no GNR domain coarsening is observed. This is further emphasized by row Fig. 8(b), where the skeletonization was carried out exactly as for Fig. 6(b). Note, while the domain size stays largely unchanged, the nanoparticle orientations do not remain constant in time. Rather, some flipping of GNRs orientations clearly takes place.

**Figure 9:**
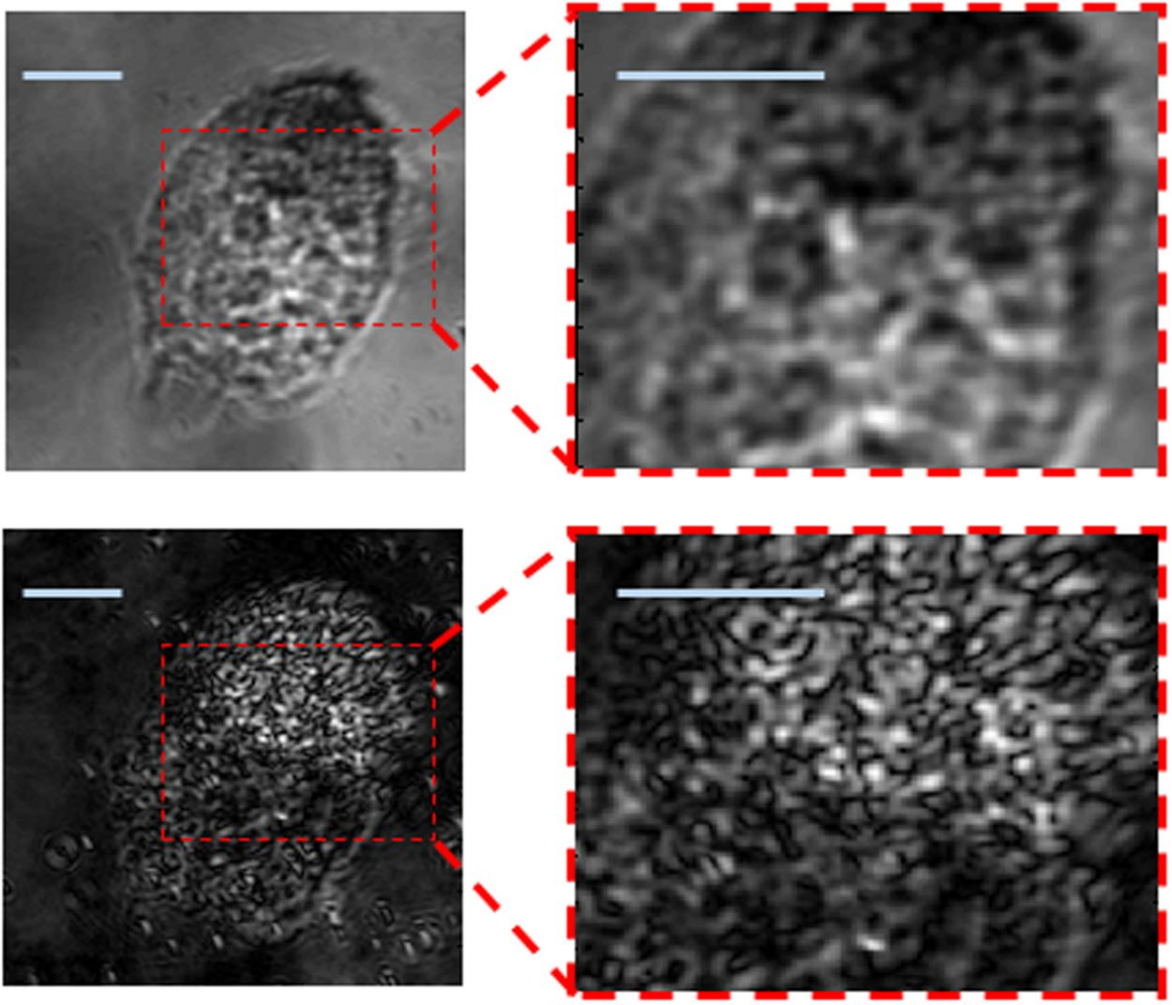
Overlapping live GNR-loaded cells are clearly distinguished by the PMLI. The suspected cells, crawling one on top of the other during the apoptosis, are virtually indistinguishable by the conventional BF microscopy (top), yet their contours are clearly visible by the PMLI (bottom). The same field of view is shown by both techniques, with the intensity values normalized for comparison. The insets on the right zoom onto the areas marked by the dashed red rectangles. The scale bars (cyan) correspond to 10 μm.

The enhanced SNR of the PMLI method allows the cells behavior during the apoptosis to be studied in a greater detail. While no true three-dimensional imaging is possible with any of these techniques, the increased SNR may partly compensate for the low resolution along the optical axis. In particular, we demonstrate that PMLI can separate between two zones, where two GNR-treated cells, are apparently crawling one on top of the other during their apoptosis. The area where an overlap between the two cells is suspected to take place is marked by a red dashed rectangle in Fig. 9. By conventional BF imaging, this area is slightly darker and has some disturbance to its right; however, the contrast is low and the darkening could definitely be attributed to an experimental noise. The much better contrast of the PMLI image allows the overlapping cells to be distinguished. The nanoparticles, which are now bright, clearly show the location and the shape of the two cells, as also their overlapping area (bright), where there is a higher concentration of particles.

## Conclusions

We have demonstrated that the image contrast, the SNR, and the resolution in either the BF or the DF imaging modes, can be dramatically enhanced by inducing a periodic signal flickering, through the manipulation of light polarization in samples labeled by anisotropic nanoparticles. We have shown that the flickering enables a more accurate determination of GNR positions and orientations, allowing for the differentiation between neighboring GNRs, which could not be resolved by the conventional optical methods. Implementing our techniques with either a DF or a BF microscopy setup, we have demonstrated its capability in imaging of the individual GNRs, as also in following the dynamics of large collections of GNR labels, in live biological cells. Future PMLI studies, exploiting particles with different geometries or stained with fluorescent fluorophores may further enhance the SNR values and add additional capabilities to the method. The strong optical anisotropy of the GNRs allows the imaging to be carried out not only in optically-isotropic media, but also in samples where a weak optical anisotropy is present, like in the currently-studied biological cells. Our technique is readily applicable to most existing optical microscopy systems, with only minor and non-expensive modifications needed. Not requiring any expensive equipment makes this method to be highly attractive for commercial, academic, and even amateur microscopy applications.

## Methods

Imaging method. For sample labeling, we employ GNRs (A12-50-800-CTAB-25, Nanopartz Inc., Loveland, CO), ~50 nm in diameter and ~145 nm in length. The employed illumination wavelengths of 780 nm and 530 nm match the transverse and the longitudinal SPR excitation modes, respectively. The employed GNR size is typical for intracellular labeling; similar GNR dimensions were also typically adopted in studies of cell-GNR interactions^12,13,15–20^.

To selectively excite the longitudinal SPR modes of the GNRs in the DF imaging mode, we vary the polarization of the illuminating beam. In the BF mode, the whole sample was illuminated with a circularly-polarized light, exciting the transverse SPR mode of all the GNRs. In order to modulate the BF signal, we introduce a rotating polarizer right in front of the camera. Thus, the GNR-orientation-dependent SPR polarization is selected.

With either the BF or the DF imaging mode adopted, we enhance the SNR by employing the lock-in amplification technique, with the polarization rotation frequency *f*_*p*_ known from the experimental settings. Thus, the in-phase and the quadrature components are obtained:

1 $${I}_{x}=\frac{1}{N}{\sum }_{t=1}^{N}{I}_{sig}(x,y,t)cos(2\pi ft),\,{I}_{y}=\frac{1}{N}{\sum }_{t=1}^{N}{I}_{sig}(x,y,t)sin(2\pi ft)$$

where *I*_*sig*_*(x*, *y*, *t)* is the raw signal of each pixel, *I*_*x*_ and *I*_*y*_ are the “in-phase” and “quadrature” components and *f* = *2f*_*p*_, due to the two-fold symmetry of the polarization. These quantities yield the lock-in filtered signal intensity *I* and the orientation angle α of the rod, within the horizontal plane:

2 $$I(x,y)=\sqrt{{I}_{x}^{2}(x,y)+{I}_{y}^{2}(x,y)},\,\alpha =2\cdot ta{n}^{-1}(\frac{{I}_{y}}{{I}_{x}})$$

Even a very small number of rotation cycles is sufficient. For example, a dramatic 25 dB increase in the SNR is readily obtained from as few as only 5 modulation cycles^47^.

Experimental setup. To test our technique, we used two types of experimental systems - DF and BF as shown in Fig. 1(a,b), respectively. The setup in Fig. 1(a) consists of a simple DF microscope where a collimated light-emitting diode (LED) illumination (Prizmatix UHP-Mic-LED-532) passes through a patch stop, after which it is focused onto the sample by a second lens. This setup generates oblique illumination. Prior to hitting the sample, the illumination passes through a step-motor-controlled rotating polarizer. The light emanating from the sample is collected using an objective (Nikon Plan Apo λ 40x/0.95), followed by an aperture. Further lens array (not shown) magnifies (M = 60) and images the sample on a CCD (Nikon DS-fi1) camera.

A sketch of the BF setup is shown in Fig. 1(b). A linearly-polarized 785 nm CW laser beam passes through a quarter wavelength plate, to produce a circularly-polarized illumination. To eliminate speckles and interferences, the light then passes through a rotating speckle diffuser creating a partial coherent light. A set of beam expanders fits the beam onto the back focal plane of an objective (Nikon Plan Apo λ 20x). Thus, a partially-coherent beam illuminates the sample, with the imaging carried out by a high-magnification objective (Nikon E Plan 100x/1.25), mounted in front of a tube lens. For image modulation, a step-motor-controlled rotating polarizer is mounted right in front of the camera (QSI RS 0.4 ws, CCD Kodak KAF-0402ME).

Sample. To calibrate and assess the capabilities of our system we first hydrophobized the cover slips by exposing them to hexamethyldisilazane (HMDS) vapors. Next, a droplet of a dilute GNR solution was deposited onto the cover slip and washed away by deionized water, a couple of minutes later. Thus, a sparse spread of individual nanorods was formed on the cover slip. To mark the positions of the GNRs, we attached a TEM grid onto the cover slip (Maxtaform reference finder grids, Ted Pella, Redding, CA).

To demonstrate the capabilities of our method when applied to fixed biological samples, GNR-loaded B-16 cells were cultured and fixed as described elsewhere^47^. The cells were imaged prior to the experiment with a DF microscope (NIKON I-50) and a hyper-spectral camera, allowing the GNRs to be differentiated from their environment according to their unique spectral response.

Live cell imaging was carried out on the MC38 cells, cultured in a 5 mL DMEM (Dulbecco’s modified Eagle medium) formulation, containing 5% FCS (fetal calf serum), 0.5% penicillin, and 0.5% glutamine. To load the GNRs into the cells, glucosamine-coated GNRs were added to the medium, followed by 24 hrs of incubation in this medium, at 37 °C. After that, the cells were ready to be imaged by our method. As a control, we have also imaged the GNR-free MC38 cells subject to the same treatment, except for the GNRs being completely absent in the incubation medium.

For studies of cells at various stages of the apoptosis, the medium was removed, and the Petri dishes were washed with a PBS (phosphate-buffered saline) solution. Next, 5 mL of the PBS solution was added to each dish, followed by an addition of H_2_O_2_ to a concentration of 500 μM. The cells were imaged at 2, 3, 4 and 5 hours following the introduction of the H_2_O_2_.

## Supplementary information

Supplementary Material
