# Supplementary material for: Imaging of nanoparticle dynamics in live and apoptotic cells using temporally-modulated polarization
Source: Sci Rep. 2019 Feb 7;9:1650. doi: 10.1038/s41598-018-38375-9 (PMC6367359; doi:10.1038/s41598-018-38375-9)
Supplement: Supplementary file 1 — Supplementary Material [file 41598_2018_38375_MOESM1_ESM.pdf]

Supplementary Information

Article in *Scientific Reports*

Imaging of nanoparticle dynamics in live and apoptotic cells using temporally-modulated polarization

Omer Wagner, Moty Schultz, Eitan Edri, Rinat Meir, Eran Barnoy, Amihai Meiri, Hagay Shpaisman, Eli Sloutskin and Zeev Zalevsky

1. SI-1: Applying PMLI on fixed B16 cells – Bright-field (BF) mode

Measurements on fixed B16 cells, labeled by GNRs, implementing the PMLI method in the BF imaging mode are shown in Fig. S1. Two cells are shown, each is imaged by both the conventional BF imaging (a, c) and by the PMLI (b, d). The areas magnified in the bottom panels, are marked by red rectangles in the lower-magnification images on top. The magnified images demonstrate the dramatic improvement in the sharpness, upon application of the PMLI method.

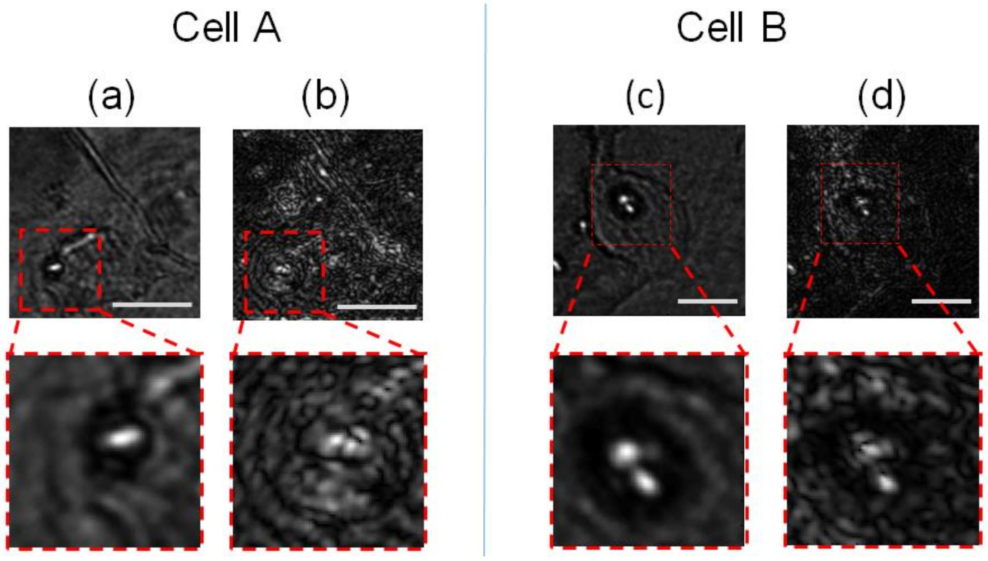

Figure S1. **The contrast and the resolution of bright-field images of GNR-labeled B-16 fixed cells are enhanced by PMLI.** The images demonstrate that a much sharper contrast is achieved with the PMLI method. Two GNR-free cells are shown. The cells are imaged without (a,c) and with (b,d) the application of the PMLI, for the sake of comparison. The same field of view is chosen in (a) and in (b); another field of view is shown in (c) and (d). The areas magnified in the bottom panels, are marked by red rectangles in the lower-magnification images on top. The intensity values are normalized, to allow for a fair comparison between the images. The scale bar lengths (gray) correspond to 10 µm.

2. SI-2: Applying PMLI on living MC38 cells – control group results for our studies of apoptosis-undergoing cells

We apply our method to living cells, as a control for our studies of apoptosis-undergoing cells. Here, the apoptosis process is not initiated, and the cells reside in DMEM (see Methods). Typical images of GNR-free cells are shown in Fig. S2. Two cells are shown, each is imaged by both the conventional BF imaging (a, c) and by the PMLI (b, d).

Remarkably, a significantly better contrast and a higher SNR is achieved with the PMLI technique also for GNR-free cells. The better contrast indicates that the optical anisotropy of the intracellular compartments is sufficiently strong for the PMLI to be applicable even with no GNRs with an intense and focused illumination employed, as in the differential polarization microscopy [24–26]. However, an even better contrast is obtained for the GNR-loaded images. In this case, the contrast of the conventional, non-modulated, BF images is already quite good, and it is further enhanced by the PMLI.

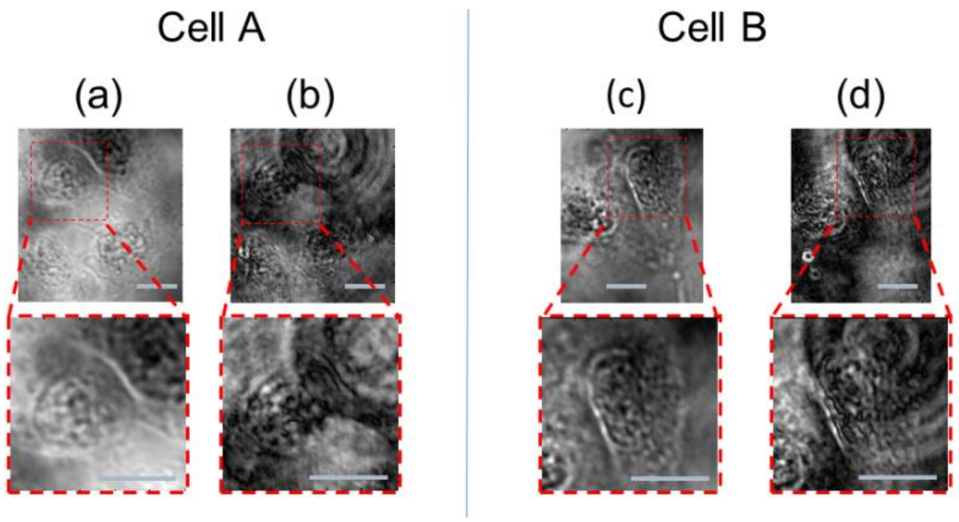

Figure S2. The contrast and the resolution of bright-field images of GNR-free live cells are enhanced by PMLI. The images demonstrate that a much sharper contrast is achieved with the PMLI method. Two GNR-free cells are shown. The cells are imaged without (a,c) and with (b,d) the application of the PMLI, for the sake of comparison. The same field of view is chosen in (a) and in (b); another field of view is shown in (c) and (d). The areas magnified in the bottom panels, are marked by red rectangles in the lower-magnification images on top. The intensity values are normalized, to allow for a fair comparison between the images. The scale bar lengths (gray) correspond to 10 μm.

3. SI-3: PMLI employing complex nanoparticles – BF mode

As mentioned in the introduction, while rods are the simplest nanoparticles which exhibit the SPR anisotropy, other more complex anisotropic nanoparticles may provide certain advantages, if used for the PMLI. In particular, the full 3D orientation of the "nanostar" particles may possibly be resolved using multiple specific illuminating wavelengths.

Here, we limit our research to a simple experimental model of the nanostars, where the SPR wavelengths are the same for all SPR polarization angles. Using our GNR sparse dispersion deposition procedure, we formed a cluster of two crossed GNRs, mimicking a very simple nanostar. The results are shown in the Fig. S3.

A SEM scan of the relevant field of view is shown in Fig. S3(a), with the image below providing a magnified view of the "nanostar"-like cluster– two rods aggregated at an angle. The bright-field image of the same area [shown in Fig. S3(b)] is unable to resolve the cluster. Our PMLI method allows the cluster to be resolved, as demonstrated in Fig. S3(c). The inset magnifies the relevant region of the sample, with the calculated GNR orientations illustrated by short blue lines, overlaid on top of the experimental image.

To the first approximation, we may separate the contributions of the two GNRs, neglecting the interactions between their plasmons. This approximation may be justified if the CTAB surfactant coating on particles' surfaces is capable to insulate the metallic contacts between the rods. In this case, each rod would respond independently, with the same frequency but with a different phase:

$$I_{rod1} = A_{rod1} cos^2(\theta + \alpha_{rod1}) = A_{rod1} \frac{1 + cos(2\theta + 2\alpha_{rod1})}{2}$$

$$I_{rod2} = A_{rod2} cos^2(\theta + \alpha_{rod2}) = A_{rod2} \frac{1 + cos(2\theta + 2\alpha_{rod2})}{2}$$

Where A_{rod1} and A_{rod2} is the coupling coefficient of each rod, θ is the polarization angle of the irradiating wave, on the image plane. α_{rod1} and α_{rod2} are the angles of the rod orientation on the coverslip plane.

The total response would be:

$$I_{total} = A_{rod1} \frac{1+cos(2\theta+2\alpha_{rod1})}{2} + A_{rod2} \frac{1+cos(2\theta+2\alpha_{rod2})}{2} = \frac{A_{rod1}+A_{rod2}}{2} + A_{total}(cos(2\theta+\alpha_{total}))$$

Where A_{total}, α_{total} are determined from $2\alpha_{rod1}$ and $2\alpha_{rod2}$. This means that the result would be periodic with a period frequency of twice the polarizer frequency - as in the simple GNR case. However, the contrast would reduce and there will be a phase shift from the original value of each of the rods.

The results presented in (c) agree with this interpretation: the particle is clearly resolved, although the contrast is poor. Also, the phase at the cluster location differs from the phase of each of the individual GNRs, as obtained by SEM.

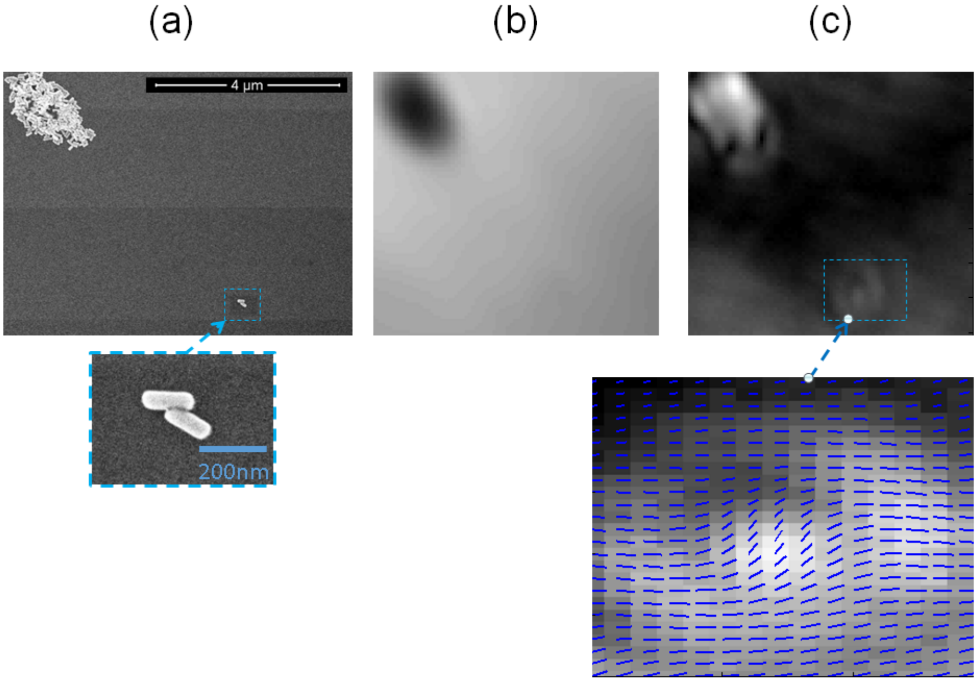

Figure S3. PMLI successfully resolves a cluster, composed of two GNRs, mimicking a nanostar particle. (a) SEM scan of the full field of view, allowing the cluster of two GNRs to be resolved. A magnified image of the cluster is shown below. (b) Conventional non-modulated BF of the same area. (c) PMLI image of the same field of view. The image below zooms onto the relevant particle location. The calculated GNR orientations are illustrated by short blue lines, overlaid on top of the magnified GNR image.
